# Transport Properties and Mechanical Features of Sulfonated Polyether Ether Ketone/Organosilica Layered Materials Nanocomposite Membranes for Fuel Cell Applications

**DOI:** 10.3390/membranes10050087

**Published:** 2020-04-29

**Authors:** Cataldo Simari, Apostolos Enotiadis, Isabella Nicotera

**Affiliations:** 1 Department of Chemistry and Chemical Technologies, University of Calabria, 87036 Rende, CS, Italy; isabella.nicotera@unical.it; 2 National Center for Scientific Research “Demokritos”, 15310 Ag. Paraskevi Attikis, Athens, Greece; aenotiadis@gmail.com

**Keywords:** sulfonated polyether ether ketone, organosilica layered materials, nanocomposite membranes, pulsed field gradient nuclear magnetic resonance (PFG-NMR), proton conductivity, dynamic mechanical analysis (DMA), proton exchange membrane fuel cells (PEM-FCs)

## Abstract

In this work, we study the preparation of new sulfonated polyether ether ketone (sPEEK) nanocomposite membranes, containing highly ionic silica layered nanoadditives, as a low cost and efficient proton exchange membranes for fuel cell applications. To achieve the best compromise among mechanical strength, dimensional stability and proton conductivity, sPEEK polymers with different sulfonation degree (DS) were examined. Silica nanoplatelets, decorated with a plethora of sulfonic acid groups, were synthesized through the one-step process, and composite membranes at 1, 3 and 5 wt% of filler loadings were prepared by a simple casting procedure. The presence of ionic layered additives improves the mechanical strength, the water retention capacity and the transport properties remarkably. The nanocomposite membrane with 5% wt of nanoadditive exhibited an improvement of tensile strength almost 160% (68.32 MPa,) with respect to pristine sPEEK and a ten-times higher rate of proton conductivity (12.8 mS cm^−1^) under very harsh operative conditions (i.e., 90 °C and 30% RH), compared to a filler-free membrane. These findings represent a significant advance as a polymer electrolyte or a fuel cell application.

## 1. Introduction

Proton exchange membranes (PEMs) have gained enormous interest for their potential application in the PEM fuel cell (PEMFC) system, which is considered as one of the most promising electrochemical devices for clean and highly efficient power generation [1,2]. Currently, Nafion^®^, a perfluorosulfonic acid (PFSA) polymer, is widely recognized as the electrolyte of choice for both hydrogen (H_2_-PEMFC) and direct methanol fuel cells (DMFC), since they can effectively conjugate high hydrolytic and oxidative stability with an excellent proton conductivity in a fully hydrated state [3,4,5,6,7]. Nonetheless, the poor mechanical stability, the loss of conductivity at temperature above 80 °C at low humidity, and the high permeability to reactants (methanol above all) are most of the severe drawbacks still hindering the large-scale commercialization of PEMFC based on PFSA membranes. Furthermore, the synthesis of perfluorinated polymers has enormous economic and environmental costs [8,9]. To the state-of-the-art, the development of alternative no-fluorinated polymers seems to represent the most promising strategy to elevate the electrochemical performance of fuel cell systems [10]. In this regard, several aromatic thermoplastics polymers are being actively investigated [11], such as polybenzimidazole [12,13,14], polyphenylsulfone [15,16,17], polyether ether ketone [18,19,20], polyether sulfone [21,22,23], polysulfone [24,25,26], etc. In such a plethora of alternative aromatic polymers, polyether ether ketone (PEEK) seems to have the best potential for practical utilization as PEM, due to its balanced combination of excellent mechanical properties, superior thermo-oxidative stability and, obviously, its low cost [27,28]. The intrinsic hydrophobic nature of this polymer is generally surmounted by chemical modification of the polymers’ chains. Through simple electrophilic substitution, sulfonic acid functionalities can be easily incorporated onto the aromatic backbone of PEEK and the corresponding sulfonated derivatives obtained (sPEEK). De facto, sulfonation makes the polymer chains ion exchangeable, increases the solubility of the macromolecules, and aids in the transport of cations [29]. The degree of sulfonation (DS) can be controlled by reaction time and temperature [30] and is a crucial focus for the performance of sPEEK [31]. In principle, the proton transport of sPEEK polymer is lower in comparison with Nafion, mainly because the peculiar microstructure does not favour the formation of continuous conducting channels. Higher sulfonation degree enhances the density of acid sites, thus facilitating the conductivity, but generally results in mechanical instabilities due to the excessive swelling. Besides, sulfonic acid groups rapidly decompose in aqueous environment, also decreasing the chemical stability of the membrane during long-term use [32,33,34]. In other words, a clear trade-off relationship exists between satisfactory conductivity and the mechanical and chemical features [35]. Therefore, further improvements are still required to overcome these big obstacles and definitively release the application of sPEEK in PEMFCs. 

To solve this key issue, the blending sPEEK with non-functional polymers and/or solids blending have been largely explored to prepare composite membranes having high temperature tolerance and electrochemical performances comparable to Nafion [36,37,38,39,40,41]. Despite this, the development of organic–inorganic composites membranes seem to date the most promising strategy [42]. Various inorganic nanofillers, such as tungsten oxides [43], silica [44,45], sulfonated silica [46], zirconium phosphate [47], Fe_2_TiO_5_ [48], boron phosphate [49], tin oxide [50], Ba0.9Sr0.1TiO_3_ [51], sulfonated core-shell TiO_2_ [52] and heteropolyacids [30], have been effectively incorporated in the sPEEK matrix producing membranes with enhanced dimensional stability and reduced permeability toward methanol, without compromising the proton conductivity. Unfortunately, these materials generally tend to agglomerate in the polymer matrix [53] or even dissolve in water present in the membrane [30], prejudicing the chemical stability and durability of the corresponding PEM. Despite this, very few studies have investigated the use of 2D plate-like layers as additives in the sPEEK matrix, such as graphene oxide, clays, layered double hydroxides (LDH), etc. [54,55,56,57]. However, it has demonstrated the exfoliation of hydrophilic lamellar-type nanomaterials inside Nafion matrix generally allows to prepare very homogeneous membranes exhibiting improved ionic conductivity, thermo-mechanical and chemical stability up to 130 °C [58,59,60,61]. Further, the dimensional stability and the methanol resistance could be remarkably enhanced [62,63,64,65]. 

Recently we have reported a simple and cost-effective one-pot procedure to synthesize of a new class of organosilica layered materials (SLM) decorated with a huge number of hygroscopic functional groups (sulfonated or phosphonate). Their fine dispersion in the Nafion matrix was demonstrated to produce nanocomposite membranes with remarkably improved transport properties and water retention capacity, especially at harsh conditions, as well as great enhancement in the mechanical and thermal resistances [66,67].

In the present work, we describe the sulfonation of PEEK polymer to establish the optimal sulfonation degree of the polymer. At the same time, silica layered materials (SSLM) are introduced for the preparation of efficient and low-cost nanocomposite membranes. Both pristine and nanocomposite PEMs were characterized in terms of mechanical properties, dimensional stability and proton transport behavior. In particular, the molecular dynamics of water confined to the ionic cluster of the membrane were deeply investigated by pulse field gradient (PFG) NMR technique. Finally, electrical impedance spectroscopy (EIS) was employed to probe the effect of both the sulfonation degree and the filler loading on the proton conductivity of the polymer electrolyte membranes.

## 2. Materials and Methods 

### 2.1. Materials

Polyether ether ketone (PEEK, Victrex 450PF) was purchased by ICI (London, UK) and dried in a vacuum oven overnight at 100 °C for 24 h prior to use. Sulfuric acid (95–98 wt%, Sigma-Aldrich, Milan, Italy), N,N-Dimethylacetamide (DMAc, Sigma-Aldrich, Milan, Italy) and NaOH (0.1 M, volumetric standard, Sigma-Aldrich, Milan, Italy) were of analytical grade and used as received.

### 2.2. Sulfonation of Polyether Ether Ketone

Sulfonated PEEK was synthesized according to the procedure reported by Banerjee et al. [68]. Briefly: 5 g of PEEK were solubilized in 100 mL of concentrated H_2_SO_4_ by vigorous magnetic stirring at room temperature until a homogeneous solution was obtained. Afterward, the temperature was increased at 40 °C and hold for different reaction times (3–8 h) to obtain sPEEK with different degree of sulfonation. To quench the reaction, the solution was slowly added to ice-cold distilled water under continuous stirring, resulting in the precipitation of sPEEK. The polymer flakes were filtered, washed several times with distilled water (until pH 6–7) and finally dried in a vacuum oven at 60 °C for 24 h. The obtained sPEEK polymers were denoted as sPEEK_3_, sPEEK_5_ and sPEEK_7_ accordingly to the sulfonation time, i.e., 3, 5 and 7 h, respectively. sPEEK polymer at sulfonation time higher than 7 h was water soluble and thus not used for the preparation of the corresponding PEM.

### 2.3. Synthesis of Organsilica Layered Materials

The organosilica layered materials were synthesized using a previously reported procedure [66,67]. In brief, an appropriate amount of aqueous silane solution 3-(trihydroxysilyl)propyl-1-propane-sulfonic acid (30–35% in water, Gelest) was placed in a Teflon beaker and dried to obtain a cracked and transparent xerogel monolith. Then, DI water was added and a milky suspension was created and centrifuged at 9000 rpm for 10 min. The centrifuged gel was successively washed with water (5 times) and acetone (2 times) and fine white powder is collected denoted as SSLM.

### 2.4. Membrane Preparation

Nanocomposite membranes were synthesized from sPEEK solution as follows: 1 g of sPEEK solution was prepared with DMAc until obtaining a clear solution. Various loadings of SSLM were first dispersed overnight in an adequate amount of DMAc and were then added dropwise to the solution of polymer with continuous stirring at 60 °C to ensure complete mixing. Finally, the solution was cast on a petri dish and left at 100 °C overnight, to obtain a homogenous membrane. The filler-free polymer, prepared by the same casting procedure, as well as, the composite membranes were subsequently acid-activated with a standard method, using 0.5 M H_2_SO_4_ solution and distilled water [20]. The dry thickness of all the prepared membranes was circa 50 ± 5 μm.

### 2.5. Characterization

#### 2.5.1. Ion Exchange Capacity, Sulfonation Degree and Water Uptake

Back titration method [69] was employed to determine the ion-exchange capacity (IEC, meq g^−1^) of the prepared membranes. The acid-activated membrane was exchanged in 2 M NaCl solution for 24 h to liberate the H^+^ ions. Afterward, the resulting solution was titrated using standard 0.1 M NaOH and phenolphthalein as indicator. From the volume (V_(NaOH)_) and concentration (M_(NaOH)_) of NaOH solution used to neutralize the H^+^ ions and the dry weight of the polymer (W_dry_), the IEC (meq g^−1^) was calculated as (Equation (1)):

(1) $$\mathrm{IEC}=\frac{M_{\left(\mathrm{NaOH}\right)}{\;*\;V}_{\left(\mathrm{NaOH}\right)}}{W_{\mathrm{dry}}}$$

From the IEC, the sulfonation degree (DS%) was determined by the Equation (2):

(2) $$\mathrm{DS}\left(\%\right)=\frac{M_{\left(p\right)}*\mathrm{IEC}}{1000-\left({\mathrm{IEC}*\;M}_{\left(f\right)}\right)}\;\cdot 100$$

where M*_(p)_* is the molecular weight of the PEEK repeat unit without the functional group, and M*_(f)_* is the molecular weight of the –SO_3_H group [70].

The water uptake (%) was calculated from the mass variation of the membrane in wet and dry state. The membrane, previously dried for 24 h at 60 °C, was weighted (m_dry_) and immersed in distilled water for 24 h at room temperature. After quickly blotting the surface of the film the mass in the “wet state” (m_wet_) was quickly measured. The w.u. was calculated by Equation (3):

(3) $$w.u.=\frac{m_{\mathrm{wet}}-m_{\mathrm{dry}}}{{\;m}_{\mathrm{dry}}}*100$$

Water uptake measurements ware also carried out as a function of the temperature (from 30 to 100 °C each 10 °C) by immersing the membrane in distilled water equilibrated at the selected temperature.

#### 2.5.2. NMR

^1^H NMR measurements were performed by a Bruker FT-NMR AVANCE 300 Wide Bore 7.05 T superconducting magnet (^1^H Larmor frequencies at 300.00 Hz, Bruker, Milan, Italy), equipped with Diff30 Z-diffusion 30 G/cm/A multinuclear probes with substitutable RF inserts. Here we used RF insert coils of 5 mm, selectively tuned to ^1^H. Typical 90° radio-frequency (rf) pulse lengths was circa 10 μs for protons. NMR samples were prepared according to the procedure reported elsewhere [71]. To determine the diffusion coefficients of water confined in the nanosized ionic channels of the membranes, a PFG NMR stimulated echo sequence (PGSTE) was used, as shown by Tanner [72]. In these measurements, the apparent diffusion coefficient, D, was measured at a diffusion time (Δ) of 8 ms and with a pulse length (δ) of 0.8 ms. The amplitude of the gradient pulses (*g*) varied from 50 up to 800 G/cm, incremented in 15 steps, while the echo time was 5.7 ms. The repetition time was ≥5 × T_1_, and the total acquisition time ranged from circa 1 to 5 min. D values were calculated by fitting the experimental data with the corresponding exponential function. The uncertainty in the self-diffusion measurements is ~3%. Measurements were carried out by increasing the temperature step by step from 20 to 130 °C, with steps of 20 °C and leaving the sample to equilibrate for at least 15 min at each temperature.

#### 2.5.3. Proton Conductivity

Rectangular shaped samples were directly cut from each membrane, sandwiched between two carbon black electrodes and placed in a homemade two-electrode cell [73]. This latter was fitted between the anode and cathode flow field of a fuel cell test hardware (850C, Scribner Associates Inc., Southern Pines, NC, USA). Impedance spectra were collected in the frequency range 1 Hz–1 MHz with a peak-to-peak voltage of 10 mV by using a PGSTAT30 potentiostat/galvanostat/FRA module and the resulting data analyzed by a Metrohm Autolab NOVA software. A humidification system (Fuel Cell Technologies, Inc., Albuquerque, NM, USA) allowed to control temperature and RH of the cell finely. The through-plane proton conductivity was measured as a function of the relative humidity (RH, 20–95%) in the temperature range 30–120 °C. Samples were equilibrated at least 30 min before each measurement.

#### 2.5.4. Dynamic Mechanical Analysis

The mechanical properties of the sPEEK-based membranes were evaluated by using a Metravib DMA/25 analyzer equipped with a shear jaw for film clamping. Rectangular shaped sample (35 mm × 10 mm) was subjected to dynamic stress of amplitude 10^3^ at 1 Hz in the temperature range 25–300 °C at a heating rate of 2 °C min^−1^. To collect the spectra, a periodic sinusoidal displacement was applied to the sample, and the resultant force was measured. From the ratio of loss (E’’) to storage (E’) moduli the damping factor (tan δ) was obtained, with T_g_ as the peak of tan δ versus temperature [74]. For the stress–strain test, the sample was clamped on the tensile module with a separation of 10 mm. The speed rate was fixed at 0.2 mm min^−1^. From the tensile stress versus strain plots the maximum tensile strength and the maximum strain were obtained. The investigation of films with comparable thickness (ca. 50 μm) allowed a more accurate comparison among of the resulting mechanical performances.

## 3. Results and Discussion

### 3.1. Effect of the Sulfonation Degree on sPEEK Membrane Properties 

**Figure 1:**
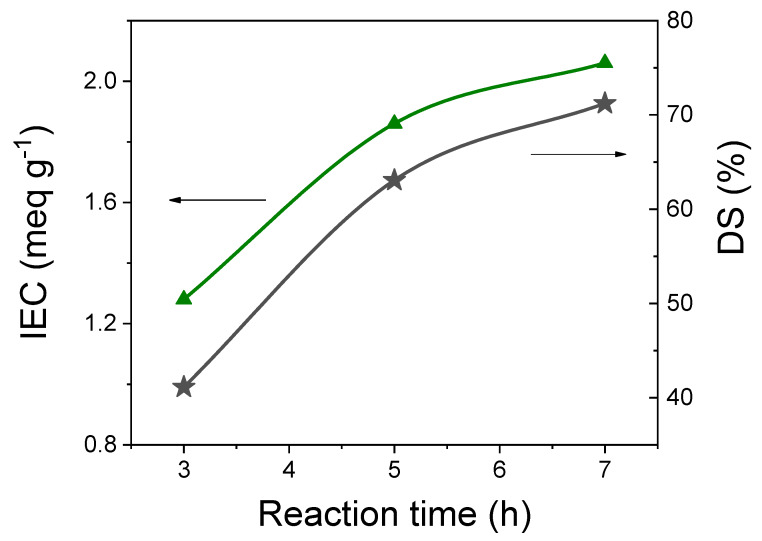
Effect of the reaction time on the IEC (meq g^−1^) and DS (%) of sPEEK.

For the sulfonation of PEEK, concentrated sulfuric acid was used as sulfonating agent. This allows for an easier and homogeneous electrophilic substitution and prevents degradation and side reactions (cross-linking) occurring with other sulfonating agents, e.g., H_2_SO_4_ or cholorosulfonic acid [18]. The effect of sulfonation time on ion exchange capacity and sulfonation degree of sPEEK membranes is shown in Figure 1, in the range of 3–7 h. Further, the preparation conditions and the main properties of each membrane are summarized in Table 1. As expected, both the IEC and DS values gradually increased with the sulfonation time, indicating a more significant number of hydrophilic domains (-SO_3_H groups) in the polymer chain. The increase rate apparently slows down over 5 h, likely suggesting the reaction is approaching to saturation. Nevertheless, after 7 h the IEC reached a value of 2.06 meq g^−1^ corresponding to an SD of 71%. It has been widely demonstrated the IEC of sPEEK is a crucial parameter since it determines the physicochemical properties of the resulting PEM. For instance, high proton conductivity is generally achieved at high IEC. Unfortunately, high DS values lead to poor mechanical properties and excessive swelling of the membranes that are undesirable in a fuel cell application. In a nutshell, the membranes should provide adequate ionic conductivity but also mechanical and hydrolytic stability to get satisfactory performance from the fuel cell. Following, a detailed study on the structure-performance relationship for several sPEEK membranes will be provided. To designate sPEEK of different IECs, the following nomenclature will henceforth be used: the sulfonation time (in hours) will follow as subscript sPEEK to discriminate one from the other, e.g., the sample obtained after 5 h of sulfonation will be referred to as sPEEK_5_.

**Figure 2:**
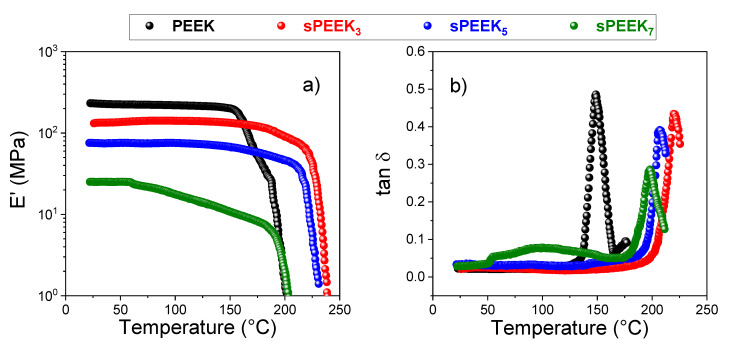
Temperature evolution of the storage moduli (**a**) and dumping factor (**b**) of PEEK and sPEEK membranes at different sulfonation degrees.

From the polymer at different sulfonation degrees, the corresponding sPEEK membranes were prepared by a simple solvent casting procedure. Subsequently, their mechanical performance was investigated by DMA analysis in a wide range of temperatures, from 20 to 250 °C. The resulting storage modulus trends (E’) and the dumping factor (tan δ) for each sample are shown in Figure 2a,b, respectively. PEEK exhibited the highest storage modulus among the investigated samples, i.e., 231.3 mPa. Further, its mechanical strength remains stable up to 150 °C, indicating PEEK is a highly thermostable polymer. The sulfonation reaction has a detrimental effect on the mechanical resistance of the membrane, leading to a progressive reduction of the E’. Surprisingly, the storage modulus of sPEEK_7_ displayed a quite anomalous behavior. The E’ of sPEEK_7_ not only was one order of magnitude lower than unmodified PEEK (24.1 MPa), but also starts to deteriorate at about 50 °C, while for the other sulfonated membranes this occurs almost at 175 °C. This suggests that at a very high sulfonation degree, not only the mechanical strength, but also the thermal resistance of the membrane was compromised. The trend is more visible in the dumping factor plots (Figure 2b), where the sPEEK_7_ revealed a first abrupt variation right above 50 °C, further confirming the weakening of the membrane likely due to the high DS. Finally, the peak of the glass transition temperature shifted from 150 °C, as the case of PEEK [30], till 200–220 °C depending on the DS. The intense interaction between sulfonic acid groups (physical crosslinking) restricts the segmental movement and thus increases the T_g_ values in ionomeric polymers [22,75,76].

**Figure 3:**
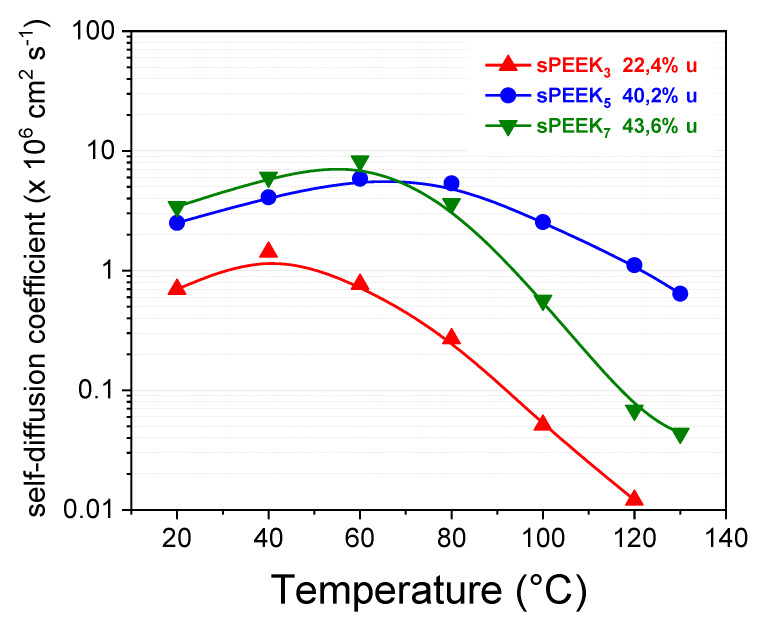
Temperature behavior of the water self-diffusion coefficients as a function of the sulfonation degree.

The transport properties of the electrolyte membranes were deeply investigated by PFG-NMR spectroscopy in a wide temperature range (20–130 °C). Figure 3 displays the temperature evolution of the self-diffusion coefficients of water confined in the hydrophilic cluster of the swollen membranes. As described in the experimental section, samples were soaked in pure water up to saturation, and thus the NMR measurements collected without any humidification system. The maximum water uptake increased from 22 wt% in sPEEK_3_ up to 44 wt% in sPEEK_7_. A similar trend, at least in the low temperature range (20–60 °C), was also observed for the diffusivity data, with the sPEEK_7_ sample showing the highest diffusion values. It should be considered that a higher DS value reflects a larger number of SO_3_H groups on the macromolecule. This could foreseeably make the polymer more hydrophilic, thus increasing the absorption capacity and the molecular dynamics of the resulting membrane. Above 80 °C, however, only the sPEEK_5_ membrane seems to preserve quite satisfactory proton mobility. The variation in DS is expected to affect the microstructures of the resulting membranes [68]. By considering the WU and the diffusivity data, it can be hypothesized sPEEK_7_ membrane has larger ionic clusters, able to absorb a higher amount of water, mainly in the bulk state (less coordinated). If from one side this provides good proton mobility by the vehicular-type transport mechanism, from the other side bulky water rapidly evaporates during heating. On the contrary, the narrower hydrophilic channels of sPEEK_5_ membrane lead to a larger amount of water molecules into “bound state”, since strongly interacting with the acidic groups of the polymer. This enables better water retention ability.

**Figure 4:**
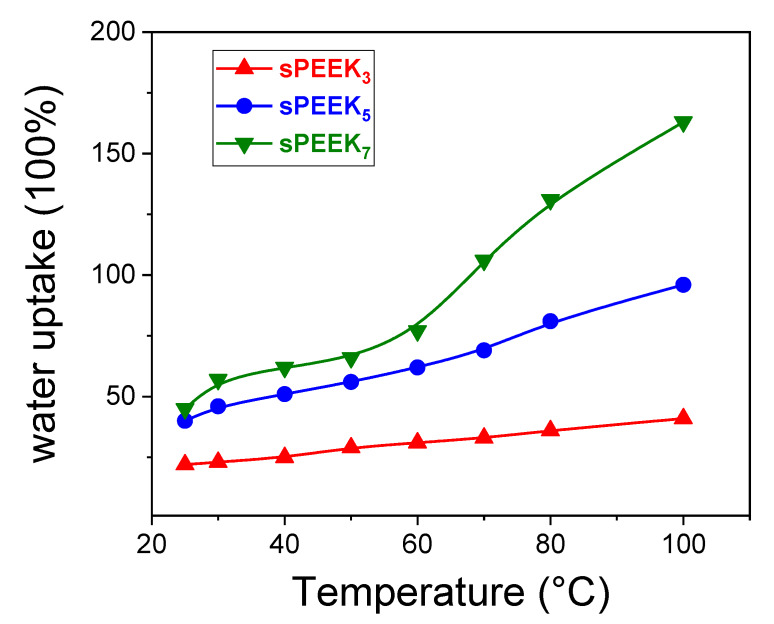
Water uptake of sPEEK membranes at different DS versus temperature, in the range 25–100 °C.

Water uptake (w.u.) is a crucial parameter in studying polymer electrolyte membranes, since directly affect most of the membrane properties. While adequate hydration level facilitates the proton transport, excessive water uptake can result in undesired membrane fragility and dimensional alteration. In the present work, the relationship between the sulfonation degree and the water uptake was also investigated from 25 °C up to 100 °C. The results were illustrated in Figure 4. As above discussed, the water uptake of sPEEK membranes is directly related to the extent of sulfonation. At low DS, the membrane displayed an almost invariable uptake with the temperature, suggesting remarkable dimensional stability. Contrarily, a huge dimensional variation has been registered for sPEEK_7_. For this latter, the water uptake at 100 °C was 3.6-fold higher than at 25 °C (163 wt% vs. 44 wt%, respectively), almost dissolving in boiling water.

**Figure 5:**
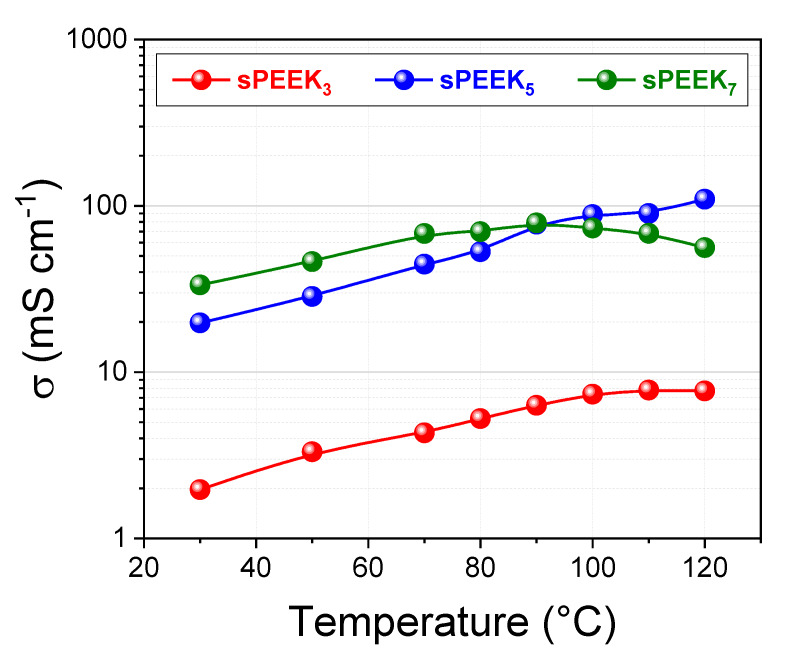
Proton conductivity of the sPEEK membranes at 90% RH.

Figure 5 illustrates the through-plane proton conductivity (σ) of the sPEEK membranes investigated at 90% RH in the temperature range 30–120 °C. Similarly to water uptake and diffusivity, the conductivity distinctly increases with DS. In particular, the σ of sPEEK_3_ membrane has found to be one order of magnitude lower in comparison with both sPEEK_5_ and sPEEK_7_. The phenomenon is strictly related to both the size and number of ionic clusters inside the membrane. At low DS, hydrophilic domains are small, highly branched and less interconnected, generating short discrete proton conduction pathways inside the PEM. The higher the sulfonation degree, the greater the size and the number of ionic clusters, favouring their overlapping and hence allowing the formation of highly interconnected proton conduction channels. Consequently, a remarkable increase in σ is registered. Further, it can be seen that σ follows almost linear increase with the temperature. In this context, the proton conductivity of sPEEK_5_ exceeds the one of sPEEK_7_ above 90 °C, reaching a value of circa 110 mS cm^−1^ at 120 °C and 90% RH. The massive swelling of sPEEK_7_ membrane under high temperatures and fully humidification conditions likely play a significant role in worsening the proton conductivity above 90 °C.

The above results clearly demonstrate that the optimization of sulfonation degree is crucial to achieve the best compromise among mechanical strength, dimensional stability and proton conductivity. Indeed, sPEEK_3_ membranes had acceptable mechanical resistance, but poor conductivities. Contrariwise, sPEEK_7_ film showed satisfactory proton conductivity, but severe structural drawbacks. For this reason, only sPEEK_5_ polymer was chosen for the preparation of nanocomposite membranes. In this regard, siliceous layered material bearing sulfonic group functionalities (SSLM) was tested as a nanofiller. The fine dispersion of SSLM in the sPEEK polymer matrix should allow preparing high performing nanocomposite electrolyte membrane.

### 3.2. sPEEK-SSLM Nanocomposite Membrane

**Figure 6:**
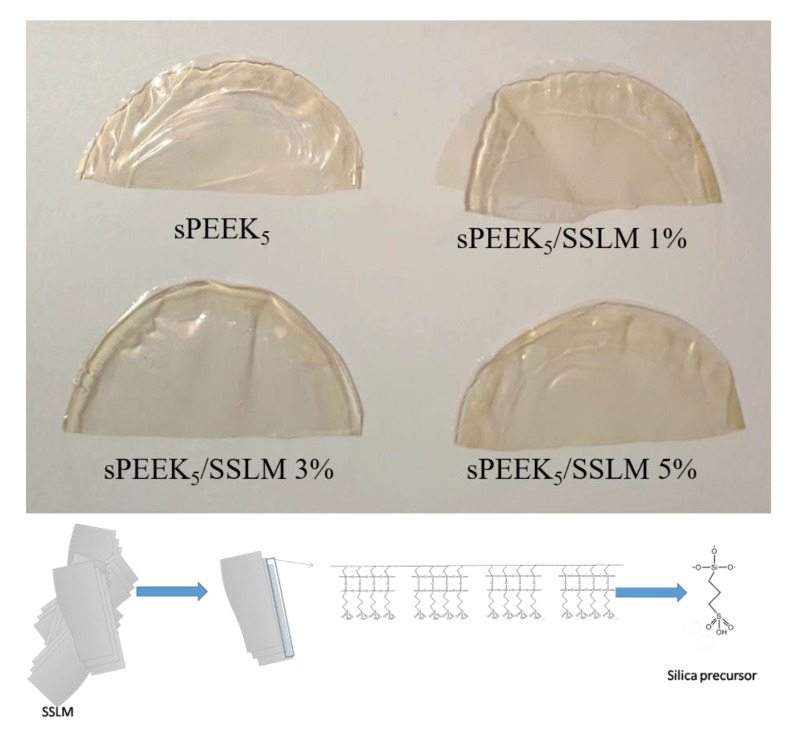
Pictures of the sPEEK_5_ and sPEEK_5_/SSLM composite membranes. The molecular structure of the sulfonated SLM is also reported.

After determining the optimum sulfonation degree, the large micron-sized plates surface decorated by oragosulfonic groups are introduced in the sPEEK_5_ matrix in order to study its effect in the final nanocomposite membranes. Figure 6 shows the pictures of the prepared membranes. With pristine sPEEK_5_, a highly transparent yellowish membrane was obtained. Upon addition of the filler, all the membrane still appears completely transparent and free of aggregates. This suggests that highly homogeneous nanocomposites were prepared also at the maximum SSLM content, i.e., 5 wt%. 

**Figure 7:**
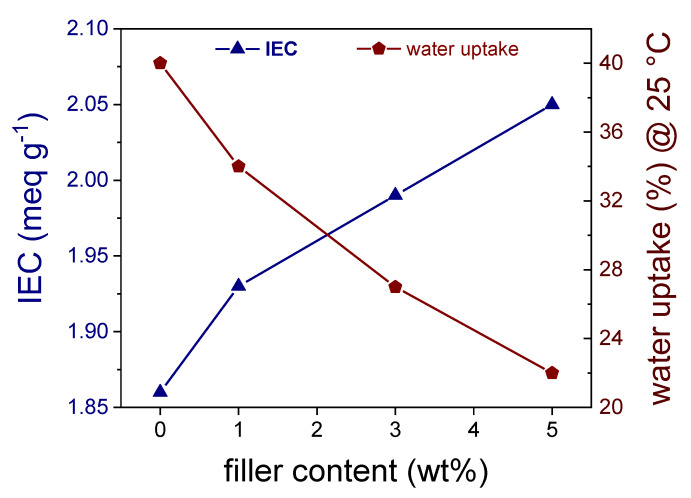
Variation of IEC and water uptake (at 25 °C) with filler loading.

The effect of filler content on the IEC and water uptake of the sPEEK composite membrane is illustrated in Figure 7. The ion exchange capacity was found to augment with the SSLM content. De facto, it increases from 1.86 to 2.05 meq g^−1^ as the filler loading grows from 0 to 5 wt%. This can be attributed to the high number of organo-sulfonic functionalities onto the SSLM surface, which trivially increases the number of exchangeable sites in the resulting nanocomposite membrane. In spite of the remarkable hydrophilic nature of SSLM particles, however, the water uptake declined significantly by increasing the nanofiller content in the sPEEK composite membranes. Indeed, at 5 wt% of SSLM, the swelling of the PEM is almost halved respect to pristine sPEEK_5_, i.e., 22 vs. 40 wt%, respectively. This result indicates the introduction of SSLM platelets somewhat decreases the volume of water channels. Supposedly, the electrostatic interaction between the surface functional groups of the organosilica lamellae and the -SO_3_H groups of the polymer restricts the mobility of the polymer chains, thus limiting the swelling ability of the membrane.

**Figure 8:**
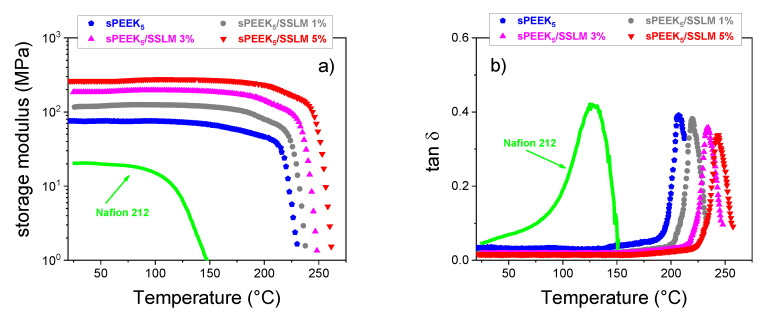
Storage modulus E’ versus T (**a**) and tan δ versus T (**b**) of sPEEK membranes at different filler content. Nafion 212 is also reported for comparison.

This also affects the mechanical properties of the membranes. Figure 8a,b illustrates the temperature evolution of the storage modulus and the tan δ, respectively, for the sPEEK-based membranes, compared with commercial Nafion 212 membrane. The strong interaction between the polymer chains and the filler remarkably increases the robustness of the PEM. The E’ of the composites increases with the filler loading and reaches the value of 260 MPa in the sPEEK_5_-SSLM 5 wt% sample, which is 3.5-fold higher than pristine sPEEK_5_ and one order of magnitude higher than Nafion 212. Similarly, the thermal resistance of the membrane notably increases upon the introduction of the filler. With 5 wt% of SSLM material, the T_g_ of the resulting membranes was upward shifted up to circa 40 °C. It is worth noting that such nanocomposite exhibit a much wider thermal stability in comparison with Nafion 212, which has a T_g_ of ca. 125 °C. Accordingly, it can effectively tolerate higher working temperature and more severe stress conditions.

**Figure 9:**
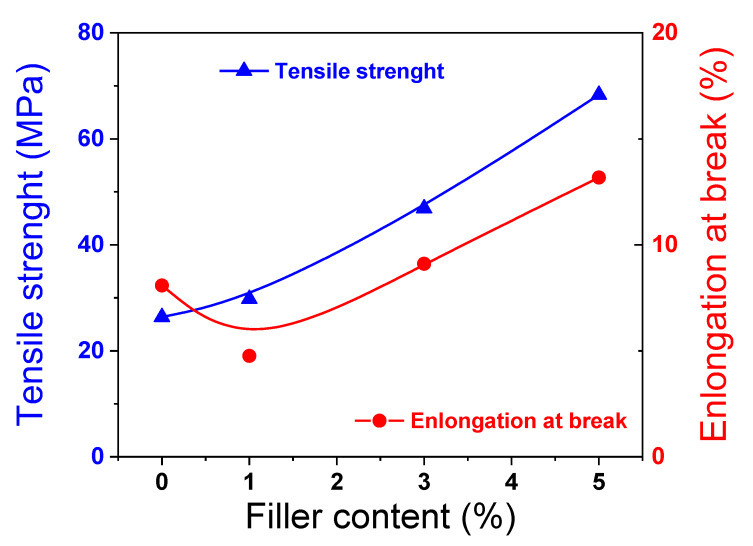
Tensile strength and elongation at break of _S_PEEK and nanocomposite membranes at 50 °C.

The stress−strain behavior of the membranes was also investigated. Figure 9 shows the tensile strength (T*_S_*) and the elongation at break (E*_B_*) data at 50 °C for different SSLM loadings. The pure sPEEK_5_ exhibited T*_S_* of 26.37 MPa and E*_B_* of 8.08%. The tensile strength was found to increase with the SSLM content. The sPEEK_5_/SSLM 5% membrane exhibited the highest tensile strength with a value of 68.32 MPa, which represents an improvement of almost 160% respect to pristine sPEEK_5_. Such a massive increase in the membrane strength is compatible with the formation of a nacre-like structure, which is typically reported for polymer matrix containing layered materials [77]. Indeed, the strong electrostatic interaction of the organosilica platelets with sPSU, in synergy with the interaction between the platelets themselves, allows for stress transfer from the polymer matrix to the high-strength SSLM nanoparticles in a manner similar to nacre [78]. Contrariwise, the elongation at break initially declined with the addition of small aliquot SSLM (i.e., 1 wt%), then raised as SSLM loading was further increased, exceeding the one of pure polymer in sPEEK5/SSLM 5% membrane. 

**Figure 10:**
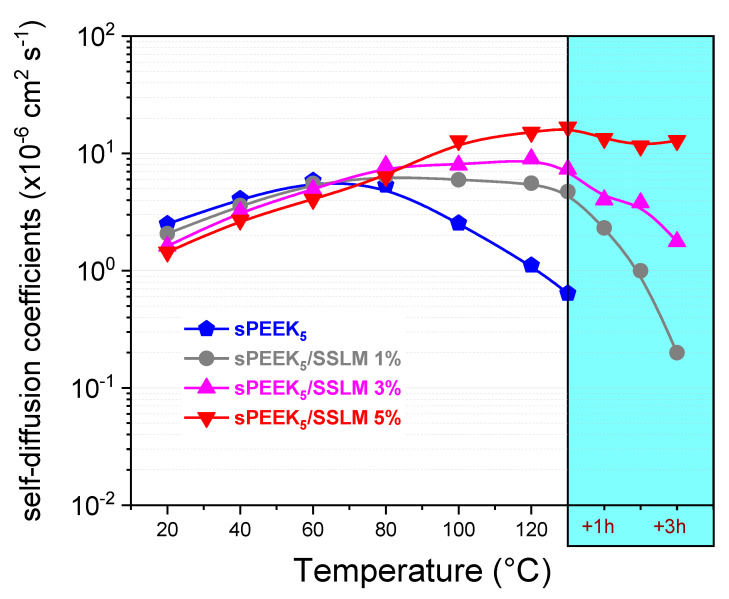
Water self-diffusion coefficients as a function of the temperature (from 20 °C up to 130 °C) for sPEEK_5_ and composites membranes. Diffusivity data collected at 130 °C after several hours are also plotted.

In terms of the water diffusion coefficient values for the nanocomposite membranes (Figure 10), it can be clearly seen that the introduction of the SSLM particles also determines the transport properties of the resulting nanocomposite membrane, in particular in the high temperature region (80–120 °C). While the diffusivity of pristine sPEEK progressively decreases above 80 °C, as a consequence of the rapid water evaporation from the membrane, D values for the SSLM-nanocomposites were found to constantly increase during heating, also exhibiting rather high values. In this regard, noteworthy are the results obtained on sPEEK_5_/SSLM 5% membrane, whose diffusivity (about 1.6 × 10^−5^ cm^2^ s^−1^) remained almost unchanged after several hours at 130 °C. This finding clearly indicates the introduction of an appropriate amount of SSLM remarkably improves the retention capacity of the membranes, being able to retain a fair amount of mobile water under very high temperatures, for a long time and without the need for an external humidification system. We would like to point out that PEM is able to effectively operate under drastic operative conditions (i.e., high T and low RH) are desired for practical fuel cell application.

**Figure 11:**
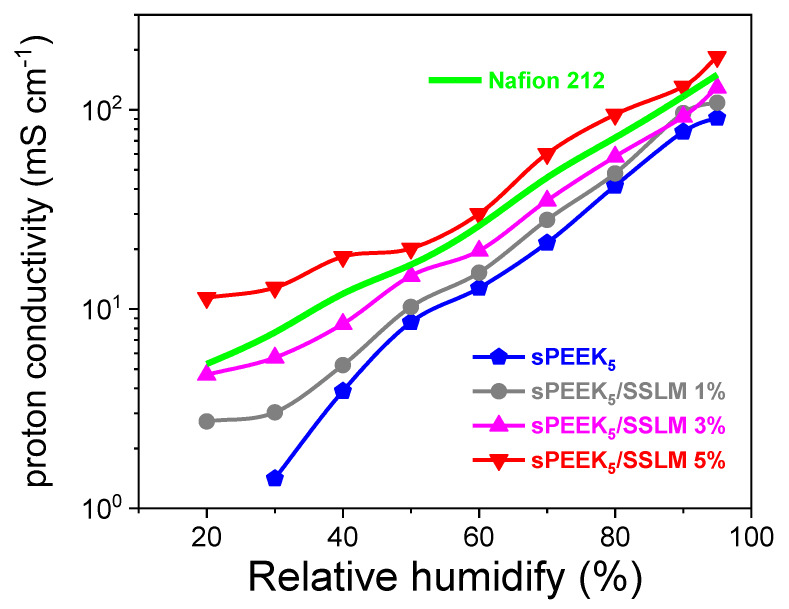
Proton conductivity (mS cm^−1^) of sPEEK membranes and Nafion 212 at 90 °C as a function of RH.

Figure 11 compares the humidity dependence at 90 °C of the proton conductivity for sPEEK membranes with Nafion 212. Trivially, the conductivity decreases with decreasing humidity, no matter which PEM was used. The conductivity of nanocomposite membranes significantly exceeds that of the sPEEK_5_ membrane, with a clear dependence from the SSLM content. The sPEEK_5_/SSLM 5% displayed highest proton conductivity over the whole RH range, surmounting also the one of Nafion 212. This reaches a value of 184.4 mS cm^−1^ at 95% RH, i.e., 2 times that of pristine sPEEK (91.0 mS cm^−1^), exhibiting one of the highest conductivities reported to date in the literature. In this regard, Table 2 reports the conductivity performance comparison of this study and analogues polymer electrolytes. The outstanding enhancement in proton conductivity upon the addition of SSLM nanoplatelets is even more evident in the lower-humidity conditions, i.e., 30% RH, where the sPEEK_5_/SSLM 5% membrane exhibited a σ of 12.8 mS cm^−1^, one order of magnitude higher compared to recast sPEEK (1.4 mS cm^−1^) and almost two times higher than Nafion benchmark (6.5 mS cm^−1^). This is obviously amenable to: (i) the higher retention capacity of this composite electrolyte; (ii) the physical crosslink provided by the SSLM platelets, that connects isolated sulfonic acid groups in dead pathways and generates continuous proton migration networks. This ensures more efficient proton conductivity via the Grotthuss mechanism, which prevails in dehydrating conditions. 

**Figure 12:**
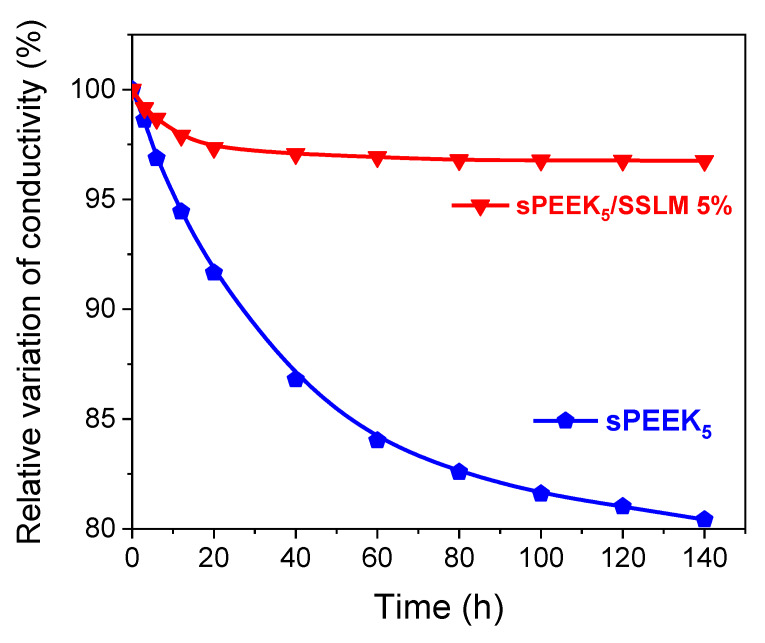
Comparison of the relative decrease of conductivity for the sPEEK and sPEEK/SSLM 5% membranes at 90 °C and 95% RH during 140 h.

To assess the hydrolytic stability of the prepared PEM, the membranes were kept at 90 °C and 95% RH for 140 h, and the decrease in their proton conductivity evaluated. As demonstrated by Li et al. [86], under such operating conditions, ignorable variation in the proton conductivity clearly corresponds to appropriate hydrolysis stability. Figure 12 shows the time evolution of the relative decrease in the conductivity for two representative samples, i.e., sPEEK_5_ and sPEEK_5_/SSLM 5%. Pristine sPEEK showed a progressive reduction in proton conductivity, reaching 20% after 140 h. The severe hydrolytic degradation of sPEEK is a well-known phenomenon, resulting from the partial decomposition of -SO_3_H groups in an aqueous environment. Contrarily, the change in proton conductivity for the nanocomposite membrane was negligible, i.e., 3.3%. The strong electrostatic interaction between SSLM platelets and sulfonic acid groups of sPEEK likely prevents the polymer backbone from degradation thus promoting the impressive increase of the hydrolytic stability exhibited by the nanocomposite.

## 4. Conclusions

We propose the preparation of sulfonated polyether ether ketone (sPEEK) nanocomposite membrane containing silica-layered materials (SLM) as low cost and efficient proton exchange membrane for fuel cell application. sPEEK at different degree of sulfonation were prepared by sulfonation of PEEK at various reaction times. Sulfonated silica nanoplatelets were synthesized through the simple sol–gel approach of 3-(trihydroxy silyl) propyl-1-propane-sulfonic acid, and composite membranes at 1, 3 and 5 wt% of filler loadings were prepared by a simple solution intercalation procedure. Both pristine and nanocomposite membranes were characterized for their mechanical, dimensional and proton transport features.

Ion exchange capacity and proton conductivity increased with the sulfonation time, however only at intermediate sulfonation degree the best compromise among mechanical strength, dimensional stability and proton conductivity was achieved. Upon introduction of SSLM materials the mechanical strength, water retention capacity, hydrolytic stability and transport properties were remarkably enhanced. sPEEK_5_/SSLM 5% membrane exhibited a tensile strength of 68.32 MPa, an improvement of almost 160% respect to pristine sPEEK. Besides, a proton conductivity of 12.8 mS cm^−1^ was recorded for this nanocomposite under very harsh operative conditions (i.e., 90 °C and 30% RH), conversely to the 1.4 mS cm^−1^ obtained in the absence of filler.

## Figures and Tables

**Table 1:** Ion Exchange Capacity (IEC), sulfonation degree (DS) and water uptake of the corresponding membranes.

Sample	Reaction Time [h]	IEC [meq g^−1^]	DS [%]	Water Uptake @ 25 °C [%]
PEEK		0	0	2
sPEEK_3_	3	1.28	41	22
sPEEK_5_	5	1.86	64	40
sPEEK_7_	7	2.06	71	44

**Table 2:** Comparison of the proton conductivity of SPEEK/SSLM 5% membrane with state-of-the-art membranes.

Membrane	Temperature [°C]	Relative Humidity [%]	Proton Conductivity [mS cm^−1^]	Ref.
sPEEK	90	95	1.4	This work
sPEEK/SSLM 5%	90	95	184	-
	90	30	13	-
sPEEK/2-AGO	120	20	11	[57]
sPEEK/DGO	120	20	3	[79]
sPEEK/TPA	100	90	95	[30]
sPEEK/WO3	100	100	19	[43]
sPEEK/TPA	100	100	95	[49]
sPEEK/sCNT	90	100	124	[80]
sPEEK/PVA@GO-NF10	90	100	70	[40]
sPEEK/PSSA-CNT	80	95	87	[81]
sPEEK/SGNF	80	95	104	[82]
sPEEK/MA3	80	100	214	[55]
sPEEK/SGO	80	30	55	[54]
**Other polymers**				
Nafion/F-GO	120	20	18	[83]
sPES/sGO	90	100	140	[84]
SPI/SPSGO-8	90	100	72	[85]
